# Auditory Stimulation Rescues Cognitive Deficit in *Fmr1*-KO Mice

**DOI:** 10.3390/brainsci16040380

**Published:** 2026-03-30

**Authors:** Mohamed Ouardouz, Amanda E. Hernan, J. Matthew Mahoney, Rodney C. Scott

**Affiliations:** 1 Nemours Children’s Hospital, 1600 Rockland Road, Wilmington, DE 19803, USA; 2 Department of Psychological and Brain Science, College of Art and Science, University of Delaware, 210 South College Street, Newark, DE 19716, USA; 3 Service of Computational Science, The Jackson Laboratory, 600 Main Street, Bar Harbor, ME 04609, USA; 4 Sidney Kimmel College of Medicine, Thomas Jefferson University, 1035 Walnut Street, Philadelphia, PA 19107, USA; 5 Great Ormond Street Hospital NHS Trust, Great Ormond Street, London WC1N 3JH, UK

**Keywords:** Fragile X Syndrome, *Fmr1*-KO mouse, auditory stimulation, recognition memory, social interaction

## Abstract

**Background/Objectives**: Fragile X Syndrome (FXS) is a neurodevelopmental disorder caused by a triplet repeat expansion in the *Fmr1* gene leading to the loss of Fragile X Messenger Ribonucleoprotein (Fmr1 protein). The loss of Fmr1 protein modulates many cell biological processes and leads to the emergence of intellectual disability and autism. FXS is modeled in *Fmr1*-KO mice that display features consistent with human FXS, including hypersensitivity, cognitive and learning deficits, hyperactivity and audiogenic seizures. Here, we investigated the effect of auditory stimulation during a range of developmental stages on recognition memory and sociability deficits in *Fmr1*-KO mice. **Methods**: *Fmr1*-KO mice were subjected to auditory stimulation for 2 min three times a day at one-hour intervals for 5 days at the nursing, juvenile and adult stages. The animals were tested for social interaction and novel object recognition at 2 to 3 months old. **Results**: During auditory stimulation, the wild running phenotype was observed in the *Fmr1*-KO juvenile animals and two animals at the nursing stage experienced status epilepticus and died. *Fmr1*-KO animals showed social deficits compared to both the control and animals exposed to auditory stimulation at the juvenile stage. In the novel object recognition task, auditory stimulation was more effective at the nursing and juvenile stages. **Conclusions**: These data show that auditory stimulation may be an effective way to restore cognitive and social deficits in FXS.

## 1. Introduction

Fragile X Syndrome (FXS) is the most common inherited cause of intellectual disability after Down Syndrome and the leading genetic contributor to autism spectrum disorder (ASD) [1]. FXS is caused by CGG trinucleotide repeat expansion exceeding 200 repeats in the *Fmr1* gene, leading to hypermethylation and functional silencing [2,3]. The resulting loss of Fragile X Messenger Ribonucleoprotein (FmrP), an RNA-binding protein that regulates the translation of a broad repertoire of synaptic proteins and coordinates multiple cell biological processes [4,5,6,7], leads to widespread synaptic dysfunction that underlies the core features of the syndrome.

The *Fmr1*-Knockout (KO) mouse is the most extensively characterized preclinical model of FXS [1], recapitulating a wide spectrum of the disorder’s hallmarks. These include physical symptoms (macro-orchidism [8]), cognitive deficits [9,10,11], behavioral impairments including changes in social interaction [12,13], repetitive behaviors [14,15], anxiety [12,16] and hyperactivity [17,18,19]. At the physiological level, *Fmr1*-ko mice exhibit dendritic spine dysmorphology [20,21,22,23,24,25] and heightened seizure susceptibility [26,27,28]. Despite considerable progress in identifying the pathophysiological mechanisms underlying these abnormal phenotypes, pharmacological interventions targeting these pathways, including mGluR5 antagonists and GABA receptors modulators, have largely failed to demonstrate clinical efficacy in human trials [29,30,31], highlighting the urgent need for alternative therapeutic strategies. Sensory stimulation has emerged as a promising non-pharmacological and non-invasive approach for treating neurological disease. In mouse models of Alzheimer’s disease, 40 Hz auditory stimulation reduces amyloid and tau buildup and improves cognition and memory [32,33,34,35,36,37,38]. In autism spectrum disorder and ADHD, random noise stimulation has been shown to enhance attention, social skills and emotional regulation and to reduce hyperactivity [39]. Directly relevant to FXS, gamma-frequency electrical stimulation of the medial septum/diagonal band of Broca in *Fmr1*-ko mice rescued social interaction, recognition memory, spatial learning and fear memory as well as normalizing basal synaptic transmission short- and long-term plasticity [40]. These findings suggest that restoring gamma-frequency neural activity may be a viable strategy for ameliorating the cognitive and behavioral deficits of FXS.

Auditory stimulation represents a particularly appealing delivery mechanism for gamma entrainment as it is non-invasive and readily translatable. In *Fmr1*-ko mice, auditory stimulation increases hippocampal gamma power [41], raising the question of whether this form of neural entrainment can produce functional cognitive benefits in this model. However, a critical complication is that high decibel auditory stimulation (~120 dB) reliably induces audiogenic seizures (AGS) in *Fmr1*-ko mice, with juvenile animals (P21-60) showing markedly greater susceptibility than adults [26,42,43]. This age-dependent seizure vulnerability mirrors the childhood-onset epilepsy that typically remits during adolescence in FXS patients [44,45]. While the cognitive consequences of AGS in *Fmr1*-ko mice have not, to our knowledge, been directly examined, early-life seizures induced by kainic acid or flurothyl in this model significantly disrupt cognitive development and social communication, with the severity and pattern of impairment varying by behavioral domain and seizure frequency [46,47,48,49,50]. These observations underscore the importance of designing auditory stimulation protocols that achieve therapeutic neural entrainment while minimizing seizure risk.

The present study was therefore designed to evaluate an auditory stimulation protocol optimized for both safety and efficacy in *Fmr1*-ko mice. We employed multifrequency white noise delivered at a moderate intensity (80 dB) to reduce AGS incidence while preserving the potential for therapeutic gamma entrainment. Social interaction and novel object were selected as primary outcome measures, given their well-established impairment in this model. We report that five days of repeated auditory stimulation was sufficient to rescue both social interaction and recognition memory in *Fmr1*-ko mice, providing proof-of-principle support for auditory stimulation as a non-invasive therapeutic strategy for FXS.

## 2. Materials and Methods

Wild-type and *Fmr1*-KO mice were obtained from the Jackson Laboratories (Bar Harbor, ME, USA). All animal work was approved by the Nemours Foundation Institutional Animal Care and Use Committee in accordance with the National Institutes of Health Guide for the Care and Use of Laboratory Animals. All animals were housed in the AAALAC-accredited Nemours Children’s Hospital Life Science Center. The animals were kept in a 12 h/12 h light/dark cycle with ad libitum access to water and food.

### 2.1. Auditory Stimulation

**Figure 1:**
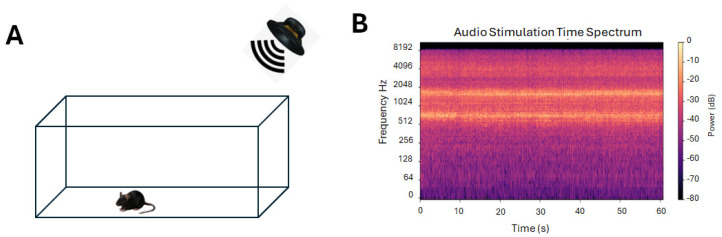
(**A**) Schematic representation of the apparatus for auditory stimulation. (**B**) Time spectrum of the auditory stimulation.

Littermate *Fmr1*-KO mice were separated into two groups. One group was subjected to auditory stimulation daily for 2 min 3 times a day at one-hour intervals for 5 days. The other group was not stimulated. Different age groups were subject to stimulation as follows: stage 1 (nursing: newborn to 21 days), stage 2 (juveniles: 22 days to 6 weeks), and stage 3 (adults: older than 6 weeks). The auditory stimulation consisted of multifrequency white noise stimulation at medium decibels (80 dB). Spectral analysis of the sound showed a Spectral Centroid (mean) of 2887.63 Hz, Spectral Rolloff (mean) of 5216.01 Hz, Spectral Bandwidth (mean) of 1980.82 Hz, and Dominant Frequency of 718.12 Hz. A Skullcandy Ounce speaker (Audio power 5 Watts, Skullcandy Inc., Park City, UT, USA) positioned 10 cm from the animal box was used to play the white noise (Figure 1). Spectral analysis of the white noise was done using Python 3.13 code (see Supplementary Material).

The mouse response to auditory stimulation was categorized as follows: no response (stage 0), wild running (stage 1) not considered as seizure (ref), tonic seizure (stage 2), clonic seizure (stage 3) and death (stage 4).

### 2.2. Behavioral Analysis

Social interaction test: A three-chamber paradigm separated by doors was used (Home made). A central empty chamber communicated through doors to two lateral chambers with a cage where a mouse can fit. After a habituation to the apparatus with no other animal for 10 min, the test mouse was put back in the home cage for 10 min before putting it back in the apparatus. During the first session, a mouse was placed in one lateral chamber and an object in the other one. The test mouse freely explored and interacted with the other mouse or the object for 10 min and was then put back in its cage for 1 h. During the second session, the object was replaced by a new animal. The test mouse freely explored and interacted with the new and familiar mouse. The exploration time and the discrimination index were calculated and compared between groups.

Recognition memory was assessed using the novel object recognition (NOR) test. After 10 min of habituation in an open field (cube or cylinder, Home made) as reported by previous work in *Fmr1*-ko mice [51,52,53], the animal was put back in its cage for 10 min. Two similar objects (glass rectangular cuboid or cylinder form) were placed in opposite sides of the arena, and the mouse was allowed to freely explore the objects for 10 min and then put back in its cage. After 1 h, one of the objects was replaced by a new one and the mouse was put back in the arena and allowed to explore for 10 min. The position of the objects differed between animals. Experiments were videotaped for later analysis. The exploration time (head of the animal toward the object at less than one cm distance, sniffing the object) and the discrimination index were calculated and compared between groups.

### 2.3. Data and Statistical Analysis

All data are presented as mean ± sem and compared using a sample Student’s *t*-test, ANOVA, using JASP software 0.19.3 version (University of Amsterdam and others). The sample *t*-test was used to determine whether the discrimination index differed from zero, and the ANOVA was used to determine whether there were differences between groups.

## 3. Results

### 3.1. Effect of Auditory Stimulation

*Fmr1*-KO mice from each litter were randomly separated into two groups; one received auditory stimulation, and the other one did not. *Fmr1*-KO mice were subject to 5 days of auditory stimulation for 2 min three times a day and were monitored for audiogenic seizures (AGS). Auditory stimulation induced death after wild running and tonic–clonic seizure in 3 animals out of 21 at development stage 1, after wild running in 6 animals out 11 at development stage 2, and no effect was observed in 10 animals at development stage 3. Wild running is not considered seizure [54,55]. Only 3 animals out of 41 animals tested at nursing stage 1 developed seizure and died.

### 3.2. Auditory Stimulation Restores Social Discrimination in Fmr1-KO Mice

**Figure 2:**
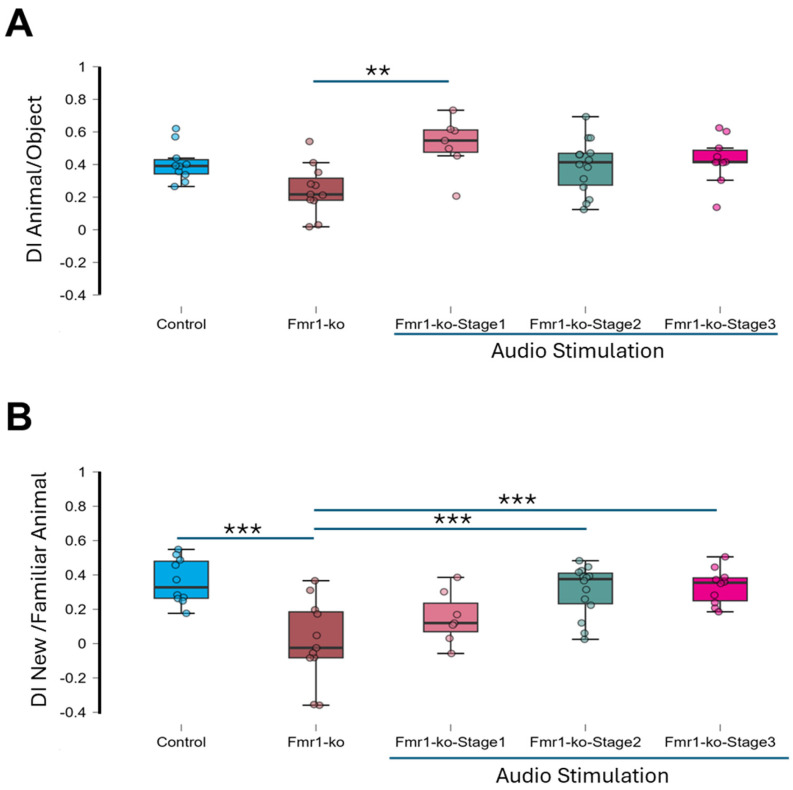
Auditory stimulation effect on social interaction. (**A**) Animal/object: All animals explore the animal more than the object (sample *t*-test *p* < 0.001). The ANOVA test showed a significant difference in the discrimination index between the 5 groups (F = 4.14, *p* < 0.006). Post hoc analysis showed a significant difference only between *Fmr1*-ko and *Fmr1*-ko-Stage1 groups (*p* < 001). (**B**): Novel/familiar animal: Only *Fmr1*-ko mice did not discriminate between the novel and familiar animal object (DI = 0.01 ± 0.07, *p* = 0.868), while all other groups did, including the control (DI = 0.41 ± 0.06, *p* < 0.001), *Fmr1*-ko-Stage1 (DI = 0.51 ± 0.06, *p* < 0.001), *Fmr1*-ko-Stage2 (DI = 0.31 ± 0.04, *p* < 0.001) and *Fmr1*-ko-Stage3 groups (DI = 0.33 ± 0.03, *p* < 0.001). ANOVA test revealed significant group differences (F = 8.71, *p* < 0.001). Post hoc analysis: The control, *Fmr1*-ko-Stage2 and *Fmr1*-ko-Stage3 mice are statistically significantly different than the *Fmr1*-ko mice (*p* < 0.001). ** *p* < 0.01 and *** *p* < 0.001; ANOVA post hoc holm test.

A three-chamber apparatus was used to assess social interaction. Animals were divided into six groups: control group (*n* = 10), unstimulated *Fmr1*-KO group (*n* = 11), and *Fmr1*-KO mice exposed to auditory stimulation at different developmental stages, namely *Fmr1*-KO-Stage1 group (*n* = 7), *Fmr1*-KO-Stage2 group (*n* = 14) and *Fmr1*-KO-Stage3 group (*n* = 10). A mouse was put in the apparatus for a 10 min habituation period and then returned to its home cage. After one hour, animals were reintroduced to the apparatus, now containing a novel mouse in one lateral chamber and an object in the other lateral chamber for 10 min (Figure 2A).

All groups explored the unfamiliar mouse more than the object, but to varying degrees, as follows: control (discrimination index (DI) = 0.41 ± 0.04, *p* < 0.001), *Fmr1*-KO (DI = 0.25 ± 0.05, *p* < 0.001), *Fmr1*-KO-Stage1 (DI = 0.52 ± 0.06, *p* < 0.001), *Fmr1*-KO-Stage2 (DI = 0.39 ± 0.04, *p* < 0.001) and *Fmr1*-KO-Stage3 (DI = 0.43 ± 0.04, *p* < 0.001). A one-way ANOVA revealed significant group differences (F = 4.14, *p* = 0.006), with post hoc tests showing that only *Fmr1*-KO-Stage1 mice performed significantly better than *Fmr1*-KO mice (*p* < 0.01, Table 1).

One hour later, to assess memory for social novelty, the object was replaced with an unfamiliar mouse (new animal). Regarding the control group (Control, DI = 0.36 ± 0.04, *p* < 0.001) and *Fmr1*-ko-Stim group, *Fmr1*-ko-Stage1 (DI = 0.15 ± 0.06, *p* < 0.05), *Fmr1*-ko-Stage2 (DI = 0.31 ± 0.04, *p* < 0.001) and *Fmr1*-ko-Stage3 (DI = 0.33 ± 0.03, *p* < 0.001) mice showed a strong preference for the new mouse while *Fmr1*-ko mice failed to discriminate between the two mice (DI = 0.01 ± 0.07; *p* = 0.87, Figure 2B). Group differences were significant (ANOVA F = 8.71, *p* < 0.001), with post hoc tests confirming impaired performance in *Fmr1*-ko mice compared to the control, *Fmr1*-KO-Stage-2 and *Fmr1*-ko-Stage3 mice but not the *Fmr1*-ko-Stage1 mice (Table 2). No sex differences were observed (ANOVA test, F = 0.006, *p* = 0.94). In the *Fmr1*-ko-Stage2 group, 6 mice out of 14 experienced wide running during auditory stimulation. To test whether wild running affects social interaction, we compared the discrimination index of *Fmr1*-ko-Stage2 mice that experienced wild running (wild running group, *n* = 6) and the one that did not (no response group, *n* = 8). The discrimination index was not different between the wild running group and no response group (animal/object: wild running DI of 0.44 ± 0.07, no-response DI of 0.35 ± 0.05, *p* = 0.27 *t*-test; new/familiar animal: wild running DI of 0.31 ± 0.06, no-response DI of 0.30 ± 0.06, *p* = 0.85 *t*-test).

These results indicate that auditory stimulation restores social discrimination in *Fmr1*-KO mice and that the effect persists for more than two weeks; behavioral testing was conducted in 2–3-month-old animals, whereas the auditory stimulation sessions occurred earlier in development, ruling out any stressful effect of the stimulation.

### 3.3. Auditory Stimulation Rescues Recognition Memory in Fmr1-KO Mice

**Figure 3:**
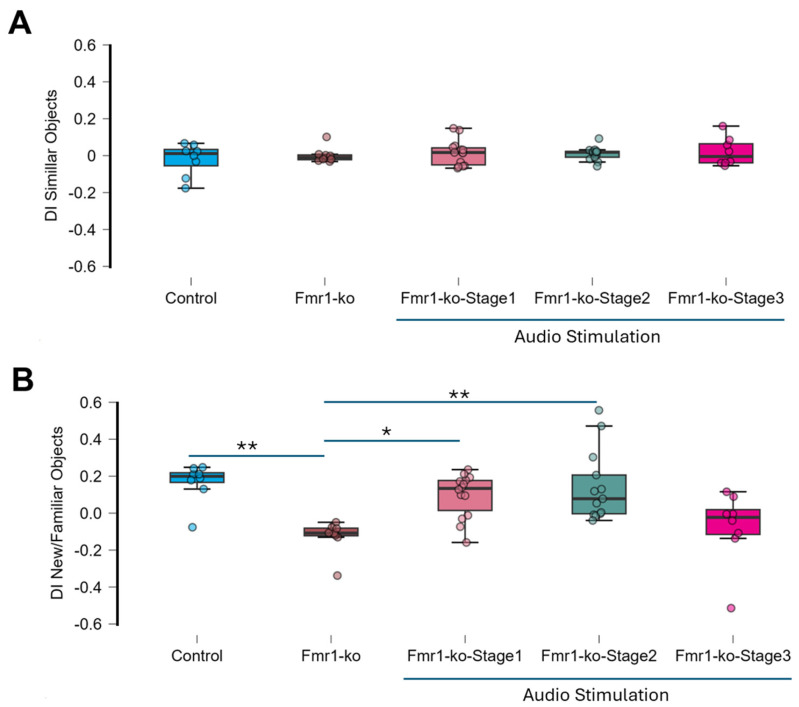
Auditory stimulation effect on recognition memory. (**A**) Similar object: All animals explore the two identical objects without preference (sample *t*-test *p* > 0.05) and there is no significant difference in the discrimination index between the 5 groups (F = 0.54, *p* = 0.707). (**B**) Novel/familiar object: The *Fmr1*-ko mice explored the familiar object more than the novel object (DI = 0.13 ± 0.03, *p* < 0.01). *Fmr1*-ko-Stege3 mice did not discriminate between the novel and familiar objects (DI = −0.08 ± 0.07, *p* = 0.314), while all other groups explored the new object more than the familiar object (control DI = 0.17 ± 0.04, *p* < 0.001; *Fmr1*-KO-Stage1 DI = 0.10 ± 0.06, *p* = 0.01; and *Fmr1*-KO-Stage2 DI 0.14 ± 0.05, *p* = 0.02). The ANOVA test shows a significant difference between groups (F = 6.94, *p* < 0.001). Post hoc pairwise comparisons revealed that the control (*p* < 0.01), *Fmr1*-ko-Stage1 (*p* < 0.05) and *Fmr1*-ko-Stage2 (*p* < 0.01) mice are statistically different than the *Fmr1*-ko mice. * *p* < 0.05, ** *p* < 0.01; ANOVA post hoc holm test.

We used the novel object recognition (NOR) task in five groups: wild-type controls, *Fmr1*-KO mice without stimulation (*Fmr1*-KO), and *Fmr1*-KO mice receiving auditory stimulation at different development stages (*Fmr1*-KO-Stage1 group, *Fmr1*-KO-Stage2 group, and *Fmr1*-KO-Stage3 group). During the training phase, all groups explored the two identical objects without preference (sample *t*-test, *p* > 0.05, Figure 3A, Table 3). The ANOVA test revealed no significant difference in the discrimination index between groups (F = 0.54, *p* = 0.707). There is no sex difference (ANOVA test, F = 0.54, *p* = 0.707).

In the test phase, conducted one hour later, one of the objects was replaced by a novel object (Figure 2B). The control group (discrimination index 0.17 ± 0.04, *p* = 0.003) and *Fmr1*-KO-Stage1 (discrimination index 0.10 ± 0.03, *p* = 0.01) and *Fmr1*-KO-Stage2 (discrimination index 0.14 ± 0.05, *p* = 0.02) mice showed a significant preference for the novel object, indicating intact recognition memory. The *Fmr1*-KO-Stage3 mice (discrimination index −0.076 ± 0.07, *p* = 0.31) did not discriminate between the two objects, and the *Fmr1*-KO mice (discrimination index—0.13 ± 0.03, *p* = 0.005) explored the familiar object more than the new object. The ANOVA test revealed a significant difference between groups (F = 6.94, *p* < 0.001), with post hoc pairwise comparisons shown in Table 4. The control (*p* < 0.01), *Fmr1*-ko-Stage1 (*p* < 0.05) and *Fmr1*-ko-Stage2 (*p* < 0.01) mice are statistically different than the *Fmr1*-ko mice.

Overall, we show here that auditory stimulation in *Fmr1*-KO animals leads to long-lasting rescue of recognition memory when done at the nursing or juvenile development stage.

## 4. Discussion

Cognitive impairment in neurodevelopmental disorder profoundly affects memory, executive function and attention among other domains, reducing the quality of life of patients and their families and caregivers. Despite advances in experimental research, translation to clinical therapeutics has remained challenging. Sensory stimulation has emerged as a promising non-invasive therapeutic strategy for neurological disease [32,33,34,35,36,37,38]. The present work extends this therapeutic framework by demonstrating that auditory stimulation can rescue cognitive impairment in a mouse model of FXS.

*Fmr1*-KO mice recapitulate many core features of FXS and represent a well-validated preclinical model for testing therapeutic interventions [1]. Here, auditory stimulation delivered at distinct developmental stages effectively rescued social interaction and recognition memory. Notably, these benefits persisted for at least two weeks or more beyond the last stimulation session, as cognitive assessments were conducted at 2–3 months of age while stimulation was given at the early development stage. This enduring effect suggests that auditory stimulation may induce long-lasting neuroplastic changes rather than simply producing transient functional improvements. However, safety considerations must be carefully addressed. During the nursing stage, 3 out of 21 animals experienced tonic–clonic seizures triggered by auditory stimulation, raising important concerns about the use of high-intensity stimulation at the early development stage. Reducing stimulus intensity may mitigate the risk and warrants systematic investigation. Notably, adult *Fmr1*-ko mice showed reduced sensitivity to high-decibel stimulation (120 dB) and did not develop AGS [26], suggesting a developmental window during which auditory stimulation exerts its greatest—but also potentially most hazardous—effects. In juvenile animals, stimulation at 80 dB successfully rescued both social interaction and recognition memory, though six out of eleven animals experienced wild running during auditory stimulation. While wild running is not classified as seizure [54,55], its occurrence nonetheless supports the recommendation to use the lowest effective stimulus intensity.

The central mechanism underlying the therapeutic potential of auditory stimulation is likely neural entrainment—the synchronization of brain oscillations by auditory stimulation [35,56,57]. Disruptions in oscillatory activity, particularly in the gamma frequency band (30–80 Hz), have been documented in *Fmr1*-ko mice [58,59,60,61], Alzheimer’s disease [56,62], Schizophrenia [63,64] and autism spectrum disorder [65,66]. Auditory stimulation at 40 Hz (gamma band) has demonstrated efficacy in Alzheimer’s disease models by reducing pathological burden and improving cognition [38]. In *Fmr1*-ko mice, gamma electrical stimulation of the medial septum/diagonal band of Broca was effective in rescuing cognitive deficit and normalized basal synaptic transmission and short- and long-term plasticity [40]. Beyond oscillatory entrainment, auditory stimulation may also engage neuromodulatory systems like cholinergic and dopaminergic pathways, thereby enhancing attention, learning and motor performance [67,68].

The observation that wild running did not negatively impact cognitive outcomes—and was in fact associated with improvement—is intriguing, though the deaths of three animals from tonic–clonic seizures underscores that the therapeutic window must be defined more precisely. These findings collectively indicate that while auditory stimulation holds genuine therapeutic promise, further work is needed to establish stimulation parameters that reliably produce cognitive benefits without adverse effects. As such, the present study should be considered a proof-of-principle demonstration, and careful caution is warranted before any extrapolation to human populations.

The broader therapeutic relevance of auditory stimulation is supported by findings across multiple neurological conditions. In stroke rehabilitation, music-supported therapy promotes cortical reorganization, probably by reinforcing sensorimotor integration and experience-dependent plasticity [69]. In epilepsy, exposure to music has been associated with reduction in seizure frequency [70,71]. Similarly, in autism spectrum disorder, music exposure was beneficial in social communication and emotional processing [72]. Together, these findings suggest that auditory stimulation engages shared neural mechanisms that extend across diagnostic boundaries.

## 5. Conclusions

Auditory stimulation rescued cognitive impairment in mouse models of FXS, with benefits persisting well beyond the stimulation period when administered during the juvenile development stage. These results support the hypothesis that auditory stimulation can restore disrupted neural dynamics and improve functional outcomes in FXS. Elucidating the precise mechanisms underlying these effects, and optimizing stimulation parameters for safety and efficacy, represents a critical next step toward translating this approach into viable clinical intervention.

## Figures and Tables

**Table 1:** *Post Hoc Comparisons*—D.I. Animal/Object.

		Mean Difference	SEM	df	*t*	*p*_holm_
Control	(*Fmr1*-ko)	0.161	0.065	47	2.468	0.138
	(*Fmr1*-ko-Stage1)	−0.117	0.074	47	−1.581	0.603
	(*Fmr1*-ko-Stage2)	0.016	0.062	47	0.261	1.000
	(*Fmr1*-ko-Stage3)	−0.021	0.067	47	−0.308	1.000
(*Fmr1*-ko)	(*Fmr1*-ko-Stage1)	−0.278	0.072	47	−3.842	0.004
	(*Fmr1*-ko-Stage2)	−0.145	0.060	47	−2.408	0.140
	(*Fmr1*-ko-Stage3)	−0.182	0.065	47	−2.784	0.069
(*Fmr1*-ko-Stage1)	(*Fmr1*-ko-Stage2)	0.133	0.069	47	1.916	0.368
	(*Fmr1*-ko-Stage3)	0.096	0.074	47	1.301	0.799
(*Fmr1*-ko-Stage2)	(*Fmr1*-ko-Stage3)	−0.037	0.062	47	−0.594	1.000

*Note. p*-value adjusted for comparing a family of 10 estimates. df: degree of freedom.

**Table 2:** *Post Hoc Comparisons*—New/Familiar Animal.

		Mean Difference	SEM	df	*t*	*p*_holm_
Control	(*Fmr1*-ko)	0.350	0.071	47	4.915	<0.001
	(*Fmr1*-ko-Stage1)	0.211	0.080	47	2.631	0.080
	(*Fmr1*-ko-Stage2)	0.055	0.067	47	0.808	1.000
	(*Fmr1*-ko-Stage3)	0.029	0.073	47	0.400	1.000
(*Fmr1*-ko)	(*Fmr1*-ko-Stage1)	−0.139	0.079	47	−1.760	0.340
	(*Fmr1*-ko-Stage2)	−0.295	0.066	47	−4.500	<0.001
	(*Fmr1*-ko-Stage3)	−0.321	0.071	47	−4.506	<0.001
(*Fmr1*-ko-Stage1)	(*Fmr1*-ko-Stage2)	−0.157	0.075	47	−2.078	0.216
	(*Fmr1*-ko-Stage3)	−0.182	0.080	47	−2.268	0.168
(*Fmr1*-ko-Stage2)	(*Fmr1*-ko-Stage3)	−0.025	0.067	47	−0.376	1.000

*Note. p*-value adjusted for comparing a family of 10 estimates. df: degree of freedom.

**Table 3:** *One Sample t-Test*—D.I. for Similar objects and D.I. New Object/Familiar Object.

	*t*	df	*p*
Control Similar Objects	−0.643	7	0.541
*Fmr1*-ko Similar Objects	0.100	7	0.923
*Fmr1*-ko-stage1 Similar Objects	0.864	13	0.403
*Fmr1*-ko-stage2 Similar Objects	1.045	12	0.316
*Fmr1*-ko-stage3 Similar Objects	0.745	7	0.481
C New Object/Familiar Object	4.485	7	0.003
*Fmr1*-ko New Object/Familiar Object	−4.013	7	0.005
*Fmr1*-ko-stage1 New Object/Familiar Object	3.006	13	0.010
*Fmr1*-ko-stage2 New Object/Familiar Object	2.679	12	0.020
*Fmr1*-ko-stage3 New Object/Familiar Object	−1.084	7	0.314

*Note.* For the Student’s *t*-test, the alternative hypothesis specifies that the mean is different from 0. df: degree of freedom.

**Table 4:** *Post Hoc Comparisons*—D.I. New Object/Familiar Object.

		Mean Difference	SEM	df	*t*	*p*_holm_
Control	(*Fmr1*-ko)	0.293	0.075	46	3.916	0.003
	(*Fmr1*-ko-Stage1)	0.071	0.066	46	1.074	1.000
	(*Fmr1*-ko-Stage2)	0.024	0.067	46	0.353	1.000
	(*Fmr1*-ko-Stage3)	0.242	0.075	46	3.234	0.015
(*Fmr1*-ko)	(*Fmr1*-ko-Stage1)	−0.222	0.066	46	−3.344	0.013
	(*Fmr1*-ko-Stage2)	−0.269	0.067	46	−4.005	0.002
	(*Fmr1*-ko-Stage3)	−0.051	0.075	46	−0.682	1.000
(*Fmr1*-ko-Stage1)	(*Fmr1*-ko-Stage2)	−0.048	0.058	46	−0.825	1.000
	(*Fmr1*-ko-Stage3)	0.171	0.066	46	2.574	0.067
(*Fmr1*-ko-Stage2)	(*Fmr1*-ko-Stage3)	0.218	0.067	46	3.246	0.015

Note. *p*-value adjusted for comparing a family of 10 estimates. df: degree of freedom.

## Data Availability

The original contributions presented in this study are included in the article/Supplementary Materials. Further inquiries can be directed to the corresponding author.

## Supplementary Materials

The following supporting information can be downloaded at: https://www.mdpi.com/article/10.3390/brainsci16040380/s1, social interaction.csv; Python code audio analysis.txt; norsound.csv.

## Abbreviations

AGS	Audiogenic seizures
ADHD	Attention-deficit/hyperactivity disorder
FXS	Fragile X Syndrome
*Fmr1*-KO	Fragile X Messenger Ribonucleoprotein 1 Knockout
